# Qualitative and quantitative alterations in intracellular and membrane glycoproteins maintain the balance between cellular senescence and human aging

**DOI:** 10.18632/aging.101540

**Published:** 2018-08-29

**Authors:** Yoko Itakura, Norihiko Sasaki, Masashi Toyoda

**Affiliations:** 1 Research Team for Geriatric Medicine (Vascular Medicine), Tokyo Metropolitan Institute of Gerontology, Tokyo 173-0015, Japan

**Keywords:** cellular senescence, human aging, intracellular glycan, lectin microarray, glycan profile

## Abstract

Glycans are associated with and serve as biomarkers for various biological functions. We previously reported that cell surface sialylated glycoproteins of dermal fibroblasts decreased with cellular senescence and human aging. There is little information on the changes in glycoprotein expression and subcellular localization during the aging process. Here, we examined intracellular glycan profiles of fibroblasts undergoing cellular senescence and those derived from aging human subjects using lectin microarray analysis. We found a sequential change of the intracellular glycan profiles was little during cellular senescence. The intracellular glycans of cells derived from aged fetus and from elderly subjects showed similar localized patterns while repeating unsteady changes. The ratio of α2-3/2-6sialylated intracellular glycoproteins in total cell extracts increased, except for a part of α2-3sialylated *O*-glycans. These findings are in contrast to those for membrane glycoprotein, which decreased with aging. Interestingly, the ratio of increasing sialylated glycoproteins in the fetus-derived cells showing cellular senescence was similar to that in cells derived from the elderly. Thus, intracellular glycans may maintain cellular functions such as ubiquitin/proteasome-mediated degradation and/or autophagy during aging by contributing to the accumulation of intracellular glycosylated proteins. Our findings provide novel mechanistic insight into the molecular changes that occur during aging.

## Introduction

*In vitro-*senescent cells are characterized by morphological changes, proliferation arrest, decreased electrophoretic mobility, and telomere shortening [1-3]. *In vivo*, senescent cells exhibit negative or positive effects such as functional disruption, hyperplasia, tumor suppression, and immune facilitation [4]. For long-term maintenance of normal biological functions, it is important to clarify protein dynamics in cells during the processes of cellular senescence and human aging.

Cell surface glycans reflect cell type as well as differentiation and other cell states [5–8]. We previously described the alterations in membrane glycosylation that occur during aging. Expression of the cell surface glycosphingolipid monosialotetrahexosylganglioside (GM1) was shown to increase with cellular senescence in human aortic endothelial cells [9], whereas the cell surface sialic acid level of glycoprotein was decreased in human dermal fibroblasts [10]. In senescent cells, the sialic acid species was identified as α2-3sialylated *O*-glycan; in human aging cells, the species was α2-3/α2-6sialylated *N*-/*O*-glycan. The above-mentioned reduction was associated with activation of fibrogenesis in human skin fibroblasts [11].

Extracellular glycans influence intracellular signaling in conjunction with extracellular sialidase, sulfatase, and deacetylase, and consequently alter the elastic fiber assembly, somite patterning, and development [12]. Maintenance of these biological functions requires repeated clearance and uptake of intracellular components, effective protein turnover, and activation of signaling pathways. Impaired clearance of glycans has been linked to various conditions such as Alzheimer’s disease and mucopolysaccharidosis. Sun et al. reported that *N*-glycoproteins in saliva increase with human aging [13]. In their study, the majority of the identified glycoproteins were acidic with low molecular weight and associated with innate immunity. The extracellular glycoproteins were more significantly increased than the intracellular ones and salivary *N*-glycoproteins related to binding, catalytic and enzymatic regulations. Accordingly, it was speculated that the intracellular glycans with biological functions that get sialylated with aging are secreted. We speculated that changes in cellular conditions during aging are reflected by intracellular glycan levels, which are influenced by multiple factors including glycan synthesis, degradation, secretion, and autophagic clearance. On the other hand, qualitative changes in the condition of glycoproteins have been used as a biomarker in cholangiocarcinoma, liver fibrosis, breast cancer, and idiopathic normal pressure hydrocephalus [14-17].

Microvesicles including exosomes—which contain both cell membrane and intracellular components—play an important role in mediating cell-cell communications. Exosomes are also a vehicle for the secretion of unwanted cellular materials through the autophagy/lysosome pathway, which is responsible for the catabolism and recycling of intracellular proteins and glycans [18]. Impaired autophagy leads to changes in lysosomal membrane proteins such as sialin and cytoplasmic accumulation of free sialyloligosaccharides [19].

The evanescent-field fluorescence-assisted lectin microarray was developed for glycoprotein profiling in various organisms [20–24] and can provide insight into the characteristics of specific cell types (e.g., stem cells) or their biological states (e.g., aging or cancer) [10, 16, 25-28 ]. In the present study, we investigated the changes in intracellular glycan profiles during cellular senescence and human aging by lectin microarray analysis using TIG human skin fibroblast lines [10].

## RESULTS

### Intracellular glycan profiles in TIG-3S, TIG-101, and TIG-102

To investigate intracellular glycan profiles associated with population doubling level (PDL) in fibroblasts derived from fetus (TIG-3S) and elderly subjects (TIG-101 and TIG-102, from 86- and 97-year-old subjects, respectively), a lectin microarray analysis was carried out in the same manner as for our previous membrane glycan profile analysis [10]. A schematic illustration of intracellular glycan profiles determined by lectin microarray is shown in Figure 1A (the list of lectins on the LecChip can be found in Supplementary Table S1). Glycan profiles of each cell line showed similar patterns in terms of the bound lectin types (Supplementary Figure S1 and Supplementary Table S2).

**Figure 1A:**
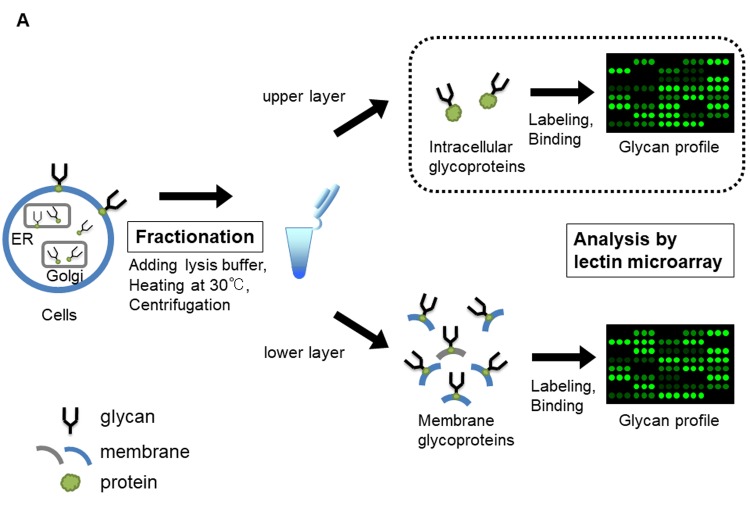
**Lectin microarray analysis for glycoproteins of TIG-3S, TIG-101, and TIG-102 at various PDLs**. Schematic illustration of lectin microarray analysis for intracellular glycoproteins (enclosed by dotted line). Collected cells were heated and centrifuged with lysis buffer, containing 0.1% protease inhibitor, followed by fractionating as intracellular glycoproteins (upper layer) and membrane glycoproteins (lower layer).

Figure 1B shows the heat map of lectin microarray signals corresponding to intracellular glycans in TIG-3S, TIG-101, and TIG-102. *N*-acetylglucosamine (GlcNAc)-oligomer-binding lectins (*Triticum vulgaris* agglutinin [WGA], *Datura stramonium* agglutinin [DSA], *Solanum lycopersicum* lectin [LEL], *Urtica dioica* agglutinin [UDA], and *Solanum tuberosum* lectin [STL] but not *Phytolacca americana* [PWM]; closed circles in Fig. 1B) and high-mannose-binding lectins (*Galanthus nivalis* agglutinin [GNA], *Narcissus pseudonarcissus* agglutinin [NPA], *Hippeastrum* *hybrid* lectin [HHL], and *Canavalia ensiformis* agglutinin [ConA]; open circles in Fig. 1B) showed the highest signal intensities in the three cell lines. In addition, the signal intensities of α2-6sialic acid-binding lectins (*Sambucus nigra* agglutinin [SNA], *Sambucus sieboldiana* agglutinin [SSA], and *Trichosanthes japonica* agglutinin I [TJA-I]; closed squares in Fig. 1B) and α1-6fucose-binding lectins (*Pisum sativum* agglutinin [PSA] and *Lens culinaris* agglutinin [LCA]; open squares in Fig. 1B) increased in all three cell lines with repeated passaging along with galactose (Gal) and high-mannose-binding lectin (*Artocarpas integliforia* agglutinin [Jacalin] and *Calystegia sepium* agglutinin [Calsepa]; closed triangles in Fig. 1B) signals. The GlcNAc-oligomer-binding lectin PWM and mannose- or complex-type *N*-glycan-binding lectin (*Tulipa gesneriana* agglutinin I [TxLC-I]; open triangles in Fig. 1B) also showed a high signal intensity, although these fluctuated with passaging. Notably, the signal intensities of TIG-3S increased over many passages (green to red in Fig. 1B), and the glycan profile of this cell line gradually became similar to that of TIG-101 and TIG-102. The lectin signals in TIG-101 and TIG-102 remained mostly unaltered over multiple passages. These results suggest that the levels of intracellular glycan-binding lectins increased with cellular senescence in TIG-3S but only altered in TIG-101 and TIG-102. The glycans in three TIG cell lines were qualitatively similar to senescent glycans. In contrast to the cell surface glycan profiles, there were no obvious differences in intracellular glycan profiles among the three cell lines except in those associated with cellular senescence.

**Figure 1B:**
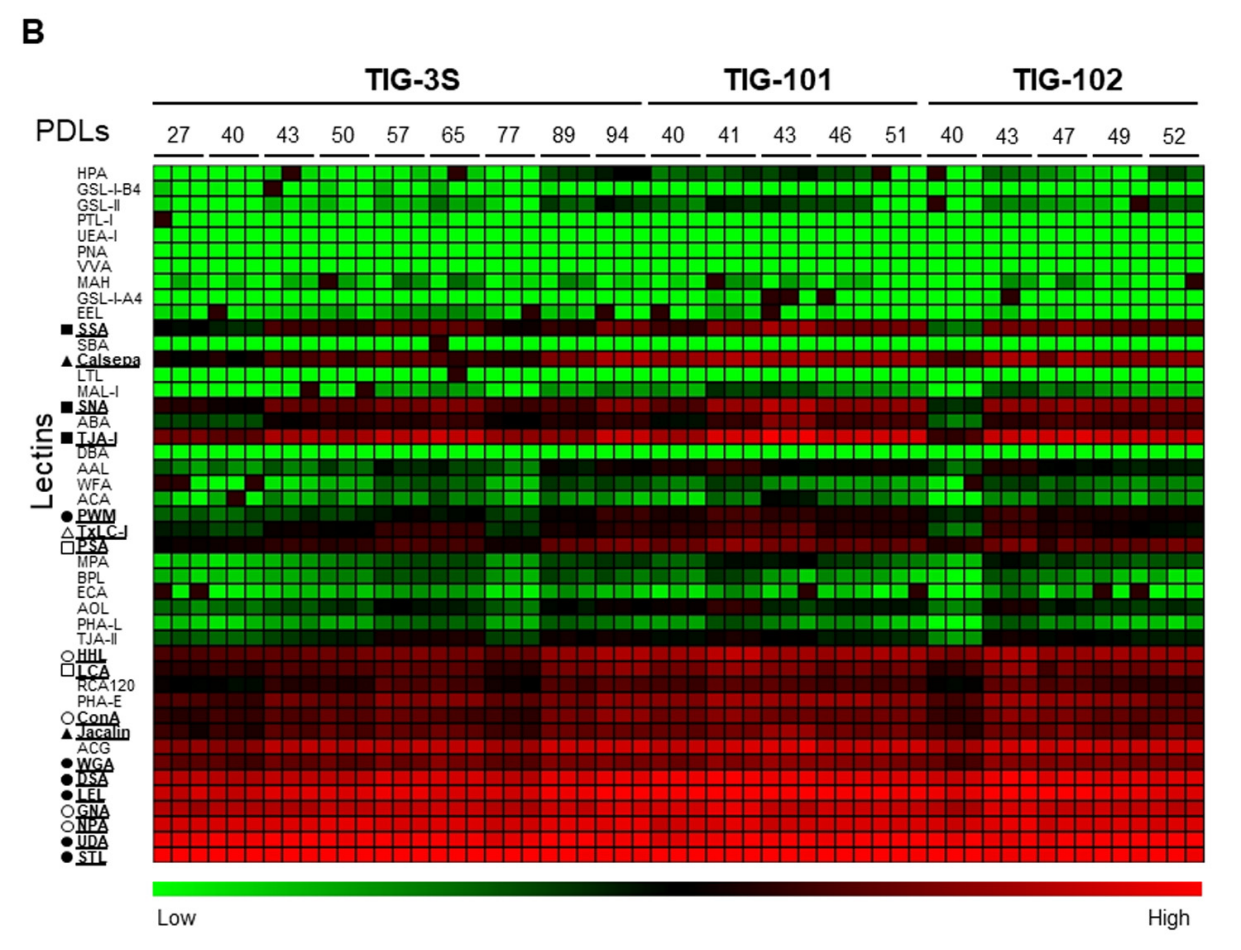
**Lectin microarray analysis for glycoproteins of TIG-3S, TIG-101, and TIG-102 at various PDLs**. Heat map of log10-transformed lectin microarray data for intracellular glycans of TIG-3S, TIG-101, and TIG-102 compared to overall lectin-binding profiles at each PDL. Rows show 45 lectins and columns show PDLs of TIG-3S, TIG-101, and TIG-102 (27–94, 40–51, and 40–52, respectively). Lectin microarray data at each PDL were obtained from triplicate measurements. The color scale indicates low (green) to high (red) ratio. Underlines for lectins are shown as specific characters. Signature indicates lectin-binding types beside lectin names (closed circle; *N*-acetylglucosamine-oligomer; open circle, high-mannose; closed square, α2-6sialic acid; open square, α1-6fucose; closed triangle, galactose or high-mannose; open triangle, mannose- or complex-type *N*-glycan).

### Changes in intracellular glycan profiles of TIG-3S, TIG-101, and TIG-102

**Figure 2:**
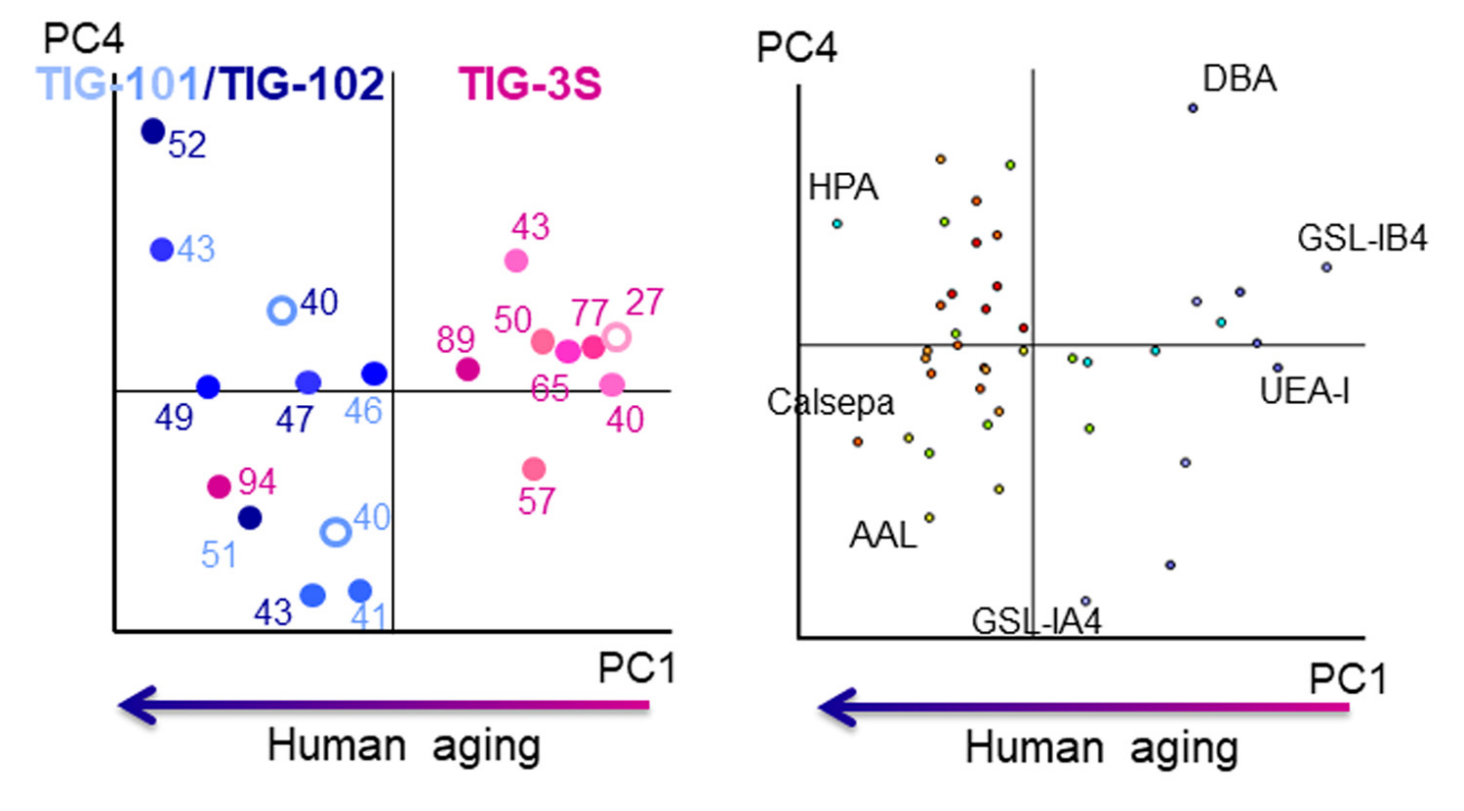
**Biplot for PCA analysis of lectin microarray data in TIG-3S, TIG-101, and TIG-102.** PC1 represents human aging. Pink, light blue, and dark blue labels represent TIG-3S, TIG-101, and TIG-102 cell lines, respectively. Color gradients (light to dark) of dots and numerals reflect progressive senescence in PDLs (i.e., young to aged). Left panel: cell passage replications, right panel: lectin replications.

There were clear differences in the membrane glycan profiles of TIG-3S and TIG-101/TIG-102 cell lines [10]. To examine in detail the correlation between changes in intracellular glycan profiles during cellular senescence and age of the cell source, we compared lectin microarray data from TIG-3S, TIG-101, and TIG-102 cell lines. A principal component analysis (PCA) was carried out with 45 lectins (Fig. 2). PC1 discriminated between TIG-3S and TIG-101/TIG-102, with clear patterns in the positive and negative directions, respectively (Fig. 2 left). Other principal components were unrelated to passage number in the three cell lines, in contrast to the cell surface glycan profiles obtained by lectin microarray analysis [10]. In fact, the plot at each PDL showed a slight trend in the negative direction on PC1 with increasing PDL, especially in TIG-3S. Only one plot for this cell line (at PDL 94) was positioned in the extreme negative direction on PC1. The plot revealed the existence of senescent cells, which was confirmed by the high PDL and cell morphology [10]. Remarkably, intracellular glycans were almost unchanged in comparison to membrane glycans. Furthermore, any lectins, which are critical for identifying alterations in intracellular glycans with aging, were not found in the bi-plot although some lectins, placed at the edge were shown (*Dolichos biflorus* agglutinin [DBA], *Helix pomatia* agglutinin [HPA] and *Griffonia simplicifolia* lectin IA4 [GSL-IA4]; GalNAc-binding lectins, *Griffonia simplicifolia* lectin IB4 [GSL-IB4]; Gal-binding lectin, *Ulex europaeus* agglutinin I [UEA-I]; α1-2fucose-binding lectin, *Aleuria aurantia* lectin [AAL]; α1-6fucose-binding lectin and Calsepa; mannose-binding lectin) (Fig. 2 right). These results suggest that there were significant differences between TIG-3S and TIG-101/TIG-102 in terms of intracellular glycan-binding levels although their overall glycan profiles appeared similar. The comprehensive glycan profile pattern and the total intensity recognized by 45 lectins at PDL 94 in TIG-3S were similar to that in the profiles of the elderly subjects in comparison to the other glycan profiles in TIG-3S. This differentiated the senescent TIG-3S (PDL 94) from other TIG-3S cells. Although the intracellular glycan composition in the three cell lines changed slightly through multiple passages unlike membrane glycans, intracellular glycan composition in the senescent TIG-3S was drastically altered and was similar to the glycan profiles of TIG-101 and TIG-102. Thus, the discrimination between TIG-3S and TIG-101/TIG-102 reflected differences in glycosylated protein dynamics with cellular senescence and human aging. Our results also indicated that there was no correlation between intracellular glycan levels and cellular senescence sequentially.

### Differences in glycan composition among TIG-3S, TIG-101, and TIG-102

**Figure 3:**
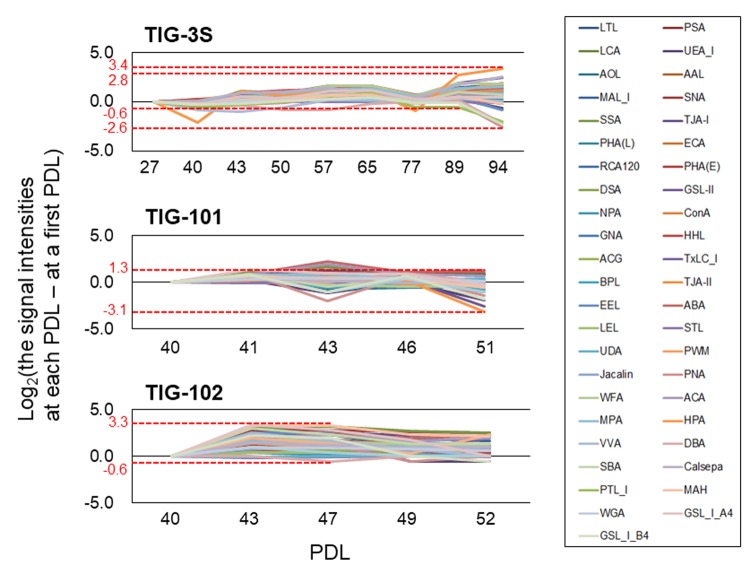
**Altered ratios of each lectin in intracellular glycans with cellular senescence.** Line graphs show differences between lectin signal intensities at various PDLs and those at the first PDL in TIG-3S, TIG-101, and TIG-102. Changes in ratio were calculated based on average signal intensity at each PDL. Highest and the lowest values of the largest change in ratio are shown for each cell line. Each lectin is shown as a different color in a box.

Discrimination of cells by PCA reflected variations in glycan composition, which could impact glycoprotein, and thus cellular functions. To clarify the dynamics of glycan composition, we compared the rates of fluctuation of lectin signals over multiple sequential passages among cell lines. The signal intensities of many lectins were positive except for TIG-101 at PDL 51. That is, many lectins increased with repeated passaging in all three cell lines relative to their first analyzed PDL (e.g., TIG-3S, TIG-101, and TIG-102 at PDL 27, 40, and 40, respectively) (Fig. 3). The maximum ratio (∆max) of the fluctuation—which represents the difference between the highest and lowest changes at each PDL—in each cell line was 6.0 for TIG-3S at PDL 94, 4.4 for TIG-101 at PDL 51, and 3.9 for TIG-102 at PDL 47 (Fig. 3 and Table 1). In addition, ∆max of the fluctuation except for senescent TIG-3S (PDL 94) was 3.3 at PDL 89. Remarkably, the variance of lectin signals in TIG-3S changed constantly over the long term, with the greatest fluctuations observed at later passages.

**Table 1:** Value of fluctuation of lectin microarray data.

	**Highest**		**Lowest**		Maximum fluctuation^^*1^^ (∆max^^*2^^)	**PDL**
**Cell lines**	**Value (Log__2__)**	**Lectin**	**Value (Log__2__)**	**Lectin**		
**Intracellular** **glycoproteins**						
TIG-3S	3.4	HPA	-2.6	PNA	6.0	94
TIG-3S except for senescent cell	2.8	HPA	-0.6	PTL-I	3.3	89
TIG-101	1.3	ABA	-3.1	HPA	4.4	51
TIG-102	3.3	MAH	-0.6	DBA	3.8	47
**Membrane** **glycoproteins**						
TIG-3S	2.0	WFA	-2.0	GSL-I-A4	4.0	94
TIG-101	1.6	GSL-I-B4	-4.4	GSL-II	6.0	43
TIG-102	3.2	DBA	-2.2	LTL	5.4	49

Highest and lowest value of the biggest changing ratio in intracellular and membrane glycoproteins of each cell line.

^^*1^^ Maximum fluctuation was calculated by ‘the highest value – the lowest value’ at each population doubling level (PDL) in each cell line.

^^*2^^ ∆max represented the maximum fluctuation

There was significantly greater variation in lectin signals for membrane glycoproteins as compared to intracellular glycans in the three cell lines at each PDL. The signal intensities of many lectins for membrane glycans tended to decrease with repeated passaging in TIG-101 and TIG-102 (Supplementary Figure S2). The maximum fluctuations in lectin signals in these cell lines were higher than those in TIG-3S—i.e., ∆max was 4.0 for TIG-3S at PDL 94, 6.0 for TIG-101 at PDL 43, and 5.4 for TIG-102 at PDL 49 (Supplementary Figure S2 and Table 1). A greater fluctuation was observed for membrane as compared to intracellular glycans except in senescent TIG-3S, which were categorized as TIG-101 or TIG-102 (Fig. 2). Lectin signals of intracellular glycans were more stable over multiple sequential passages than those of membrane glycans. These results suggest that intracellular glycan components increase slightly, whereas those of cell surface glycans fluctuate drastically during aging.

### Abundance of intracellular vs. cell surface glycans

To clarify the dynamics of intracellular and membrane glycans with aging, we compared the ratios of signal intensities of intracellular to membrane glycans for seven lectins (SNA, SSA, *Agrocybe cylindracea* galectin [ACG], *Maackia amurensis* hemagglutinin [MAH], *Erythrina cristagalli* agglutinin [ECA], PWM, and *Wisteria floribunda* agglutinin [WFA]) whose membrane glycoprotein composition changed during aging [10] in total cell extracts of each cell line (Fig. 4), and found clear changes in the abundance of intracellular and membrane glycans. As for sialic acid-binding lectins, the ratios of intracellular glycans for total lectin microarray signals in SNA and SSA were less than or comparable to those of membrane glycans in each cell line at early passages (e.g., 42%, 62%, and 41% intracellular glycans in SNA signals at PDL 27, 40, and 40 in TIG-3S, TIG-101, and TIG-102, respectively) (Fig. 4A, 4B, and Supplementary Table S3). Moreover, the ratios of intracellular glycans increased slightly with repeated passaging in the three cell lines. The proportions of intracellular glycans of TIG-101 and TIG-102 were higher than that of TIG-3S (e.g., 55%, 69%, and 68% intracellular glycans in SNA signals at PDL 94, 51, and 52 in TIG-3S, TIG-101, and TIG-102, respectively) (Fig. 4A and 4B). The ratio of intracellular glycans in the ACG (α2-3sialic acid-binding lectin) signal was less than that of membrane glycans in TIG-3S at early passages (e.g., 34% intracellular glycan at PDL 27), with the proportion of intracellular glycans gradually increasing (Fig. 4C left panel). The proportions of intracellular to membrane glycans were nearly equivalent in the total cell extract (i.e., 54% intracellular glycan at PDL 94). In TIG-101 and TIG-102, the ratio of intracellular to membrane glycans remained mostly unchanged with passaging and was equal to that of senescent TIG-3S (e.g., 56% and 55% intracellular glycans at PDL 51 and 52 in TIG-101 and TIG-102, respectively) (Fig. 4C middle and right panels). There was little change in proportion of the MAH signal over multiple sequential passages in the three cell lines, and the proportions of membrane glycans in TIG-101 and TIG-102 were much higher than that in TIG-3S (e.g., 61%, 89%, and 97% membrane glycans at PDL 27, 40, and 40 in TIG-3S, TIG-101, and TIG-102, respectively) (Fig. 4D). This result demonstrates that α2-3sialylated *O*-glycans are more abundant on membranes than intracellular glycoproteins in TIG-101 and TIG-102.

**Figure 4A-D:**
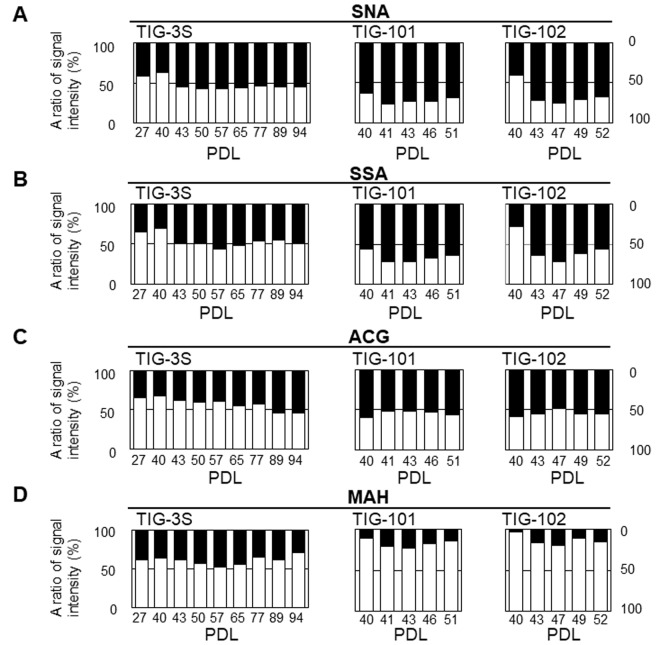
**Abundance of intracellular to cell surface glycans in seven lectins: SNA (A), SSA (B), ACG (C), MAH (D) at different PDLs.** Left, middle, and right panels show TIG-3S, TIG-101, and TIG-102, respectively. Closed and open bars represent proportions of intracellular and cell surface glycans, respectively, relative to the total array signal at each PDL. Levels of the seven selected lectins in cell surface glycans changed with aging [10].

We also compared the abundance of intracellular glycans in GlcNAc- or Gal-binding lectins to that of membrane glycans. ECA binds to lactose units (Galβ1-4Glc) and LacNAc units (Galβ1-4GlcNAc) but does not permit sialic acid residues [29]. The proportion of intracellular glycans in the ECA signal was almost the same as that of membrane glycans in TIG-3S at early passages (i.e., 45% intracellular glycan at PDL 27) (Fig. 4E left panel). The proportion of intracellular glycans oppositely to sialic acid-binding lectins decreased over multiple sequential passages (e.g., 24% intracellular glycan at PDL 94). The proportion of membrane glycans was higher than that of intracellular glycans in TIG-101 and TIG-102, and was comparable to that in senescent TIG-3S (e.g. 87% and 94% membrane glycans in TIG-101 and TIG-102, respectively, at PDL 40) (Fig. 4E middle and right panels). The change in the ratio between intracellular and membrane glycans with repeated passaging was similar between TIG-3S and TIG-101/TIG-102. The ratio of intracellular glycans in PWM—which binds not only to GlcNAc-oligomer but also to LN*n*H {Galβ1-4GlcNAcβ1-6(Galβ1-4GlcNAcβ1-3)Galβ1-4Glc} [30]—and WFA—which binds to Galβ1-3GalNAc and GalNAcβ1-4GlcNAc—showed similar trends as in ECA. The proportion of intracellular glycans decreased with passaging in TIG-3S (e.g., intracellular glycans in PWM and WFA decreased from 60% to 53% and from 31% to 20% from PDL 27 to 94, respectively) (Fig. 4F left, and Fig. 4G, left panels). The same trend was observed in TIG-101 and TIG-102. The proportions of intracellular glycans in TIG-101 and TIG-102 were lower than those of membrane glycans over multiple sequential passages (e.g., the proportion of intracellular glycans in PWM and WFA was 39% and 12% at PDL 51 in TIG-101, and 40% and 13% at PDL 52 in TIG-102, respectively) (Fig. 4F middle and right, and Fig. 4G middle and right panels). These results suggest that α2-6/2-3sialic acid residues on *N*- or *O*-glycan of intracellular glycans against membrane glycoproteins increased with cellular senescence, except that a high level of α2-3sialylated *O*-glycan (i.e., MAH) remained on the membrane in TIG-101 and TIG-102. Thus, similar proportions of glycans are altered with cellular senescence and during human aging. These quantitative changes in glycan localization were more evident in TIG-3S than in TIG-101 and TIG-102, and may reflect the aging-related accumulation of intracellular glycans.

**Figure 4E-G:**
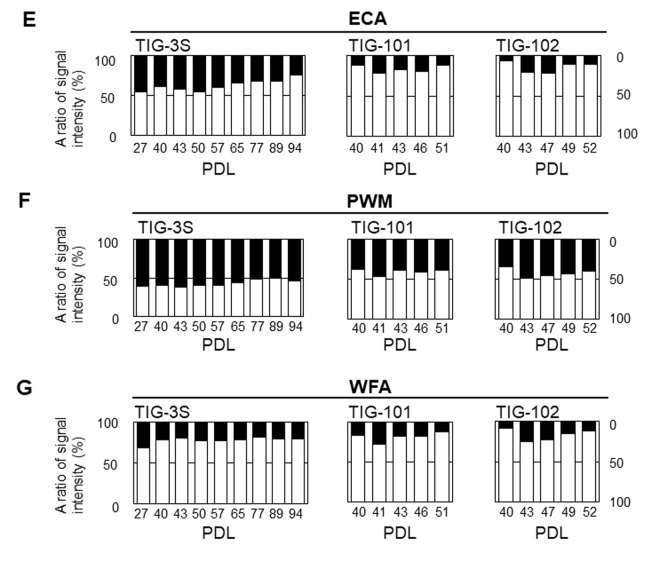
**Abundance of intracellular to cell surface glycans in seven lectins: ECA (E), PWM (F), and WFA (G) at different PDLs **. Left, middle, and right panels show TIG-3S, TIG-101, and TIG-102, respectively. Closed and open bars represent proportions of intracellular and cell surface glycans, respectively, relative to the total array signal at each PDL. Levels of the seven selected lectins in cell surface glycans changed with aging [10].

### Localization of intracellular and cell surface glycans

**Figure 5:**
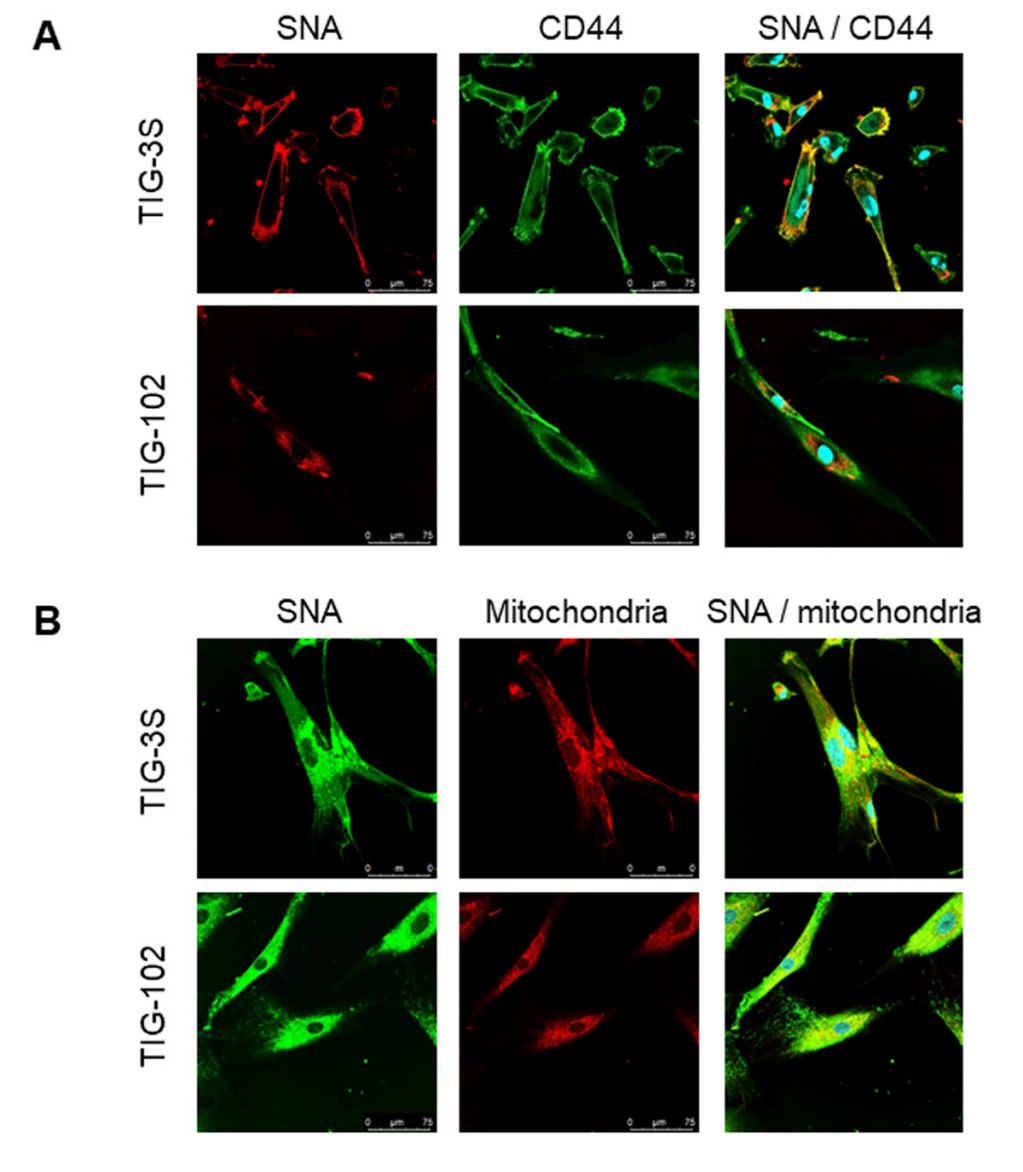
**Localization of sialylated glycoproteins in TIG cells.** (**A**) TIG-3S (PDL 38; top) and TIG-102 (PDL 46; bottom) stained with SNA (red; left panel), FITC-conjugated membrane marker (CD44, green; middle panel) and the overlay image (right panel). (**B**) TIG-3S (PDL 52; left) and TIG-102 (PDL 46; right) stained with FITC-conjugated SNA (green; left panel), an intracellular marker (mitochondria, red; middle panel), and the overlay image (right panel). Blue staining represents the nucleus.

To examine how the membrane and intracellular glycans (sialic acids), that were found to be changing by lectin microarray analysis localized within the cell, we performed double staining of TIG-3S and TIG-102 for the lectin (SNA), and membrane and intracellular location specific markers (CD44 and mitochondria, respectively). The cell-edge of TIG-3S shown in yellow was strongly stained with SNA (red) and CD44 antigen marker (green) (Fig. 5A top). It demonstrated that the sialylated proteins were highly expressed on the cell membrane. However, the cell-edge of TIG-102 was weakly stained with SNA showing reduced expression of sialylated membrane proteins in the cells derived from elderly subjects (Fig. 5A bottom). On the other hand, the intracellular space of TIG-3S shown in yellow was strongly stained with SNA (green) and mitochondrial ribosomal protein marker (red), especially at the center of the cells (Fig. 5B top), demonstrating that the sialylated proteins were mainly localized in the intracellular space. Similarly, the intracellular space of TIG-102 shown in yellow indicated the binding of SNA and mitochondria (Fig. 5B bottom). Moreover, a sporadic distribution of SNA binding-proteins was observed in the cells, suggesting that the intracellular proteins got highly sialylated and that they were present throughout the cells in the elderly subject-derived cells. Additionally, we also compared the membrane glycoproteins of younger TIG-3S (PDL 48) to aged TIG-3S (PDL 84) with SNA (red) and CD44 antigen marker (green). The cell-edge of younger TIG-3S shown in yellow was stained comparatively stronger with SNA and CD44 antigen marker than that in the aged TIG-3S (Supplementary Figure S3). These results suggest that the expression of sialylated proteins were different in the subject-derived cells and in senescence. This was in good agreement with the ratio of abundance observed by lectin microarray analysis.

### Expression of intracellular and cell surface glycoproteins

**Figure 6:**
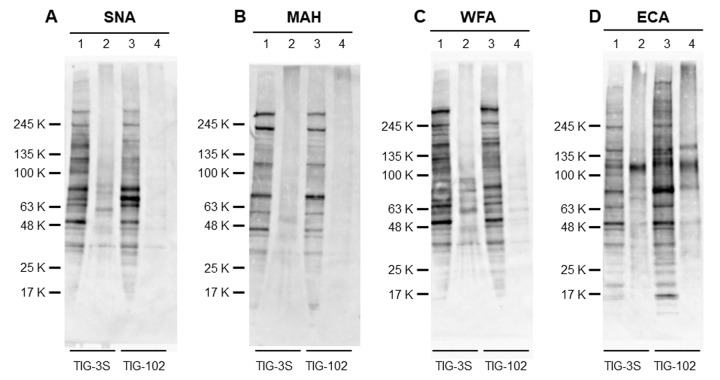
Lectin blot detection of intracellular and membrane glycoproteins of TIG cells. The intracellular extracts from TIG-3S and TIG-102 and the corresponding membrane extracts were applied to lanes 1, 3 and 2, 4, respectively. They were subjected to lectin blot analysis using (**A**) biotinylated-SNA, (**B**) -MAH, (**C**) -WFA, and (**D**) -ECA.

To compare the overall expression of intracellular glycoproteins directly with membrane glycoproteins, we analyzed the intracellular and membrane bands of TIG-3S and TIG-102 using four lectins (SNA, MAH, WFA and ECA). As for sialic acid-binding lectins, many intracellular glycoproteins of TIG-3S and TIG-102 were bound to SNA, while only a few membrane glycoproteins were bound to SNA (Fig. 6A). Although the total amount of intracellular glycoprotein bands of TIG-3S against SNA was approximately equal to that of TIG-102, some specific protein bands of TIG-102 reacted stronger than that of TIG-3S (e.g. approximate molecular weights of 70 KDa). Membrane glycoprotein bands of TIG-3S reacted strongly to SNA compared to that of TIG-102. In binding to MAH, many intracellular glycoproteins of TIG-3S and TIG-102 were bound, and only a few membrane glycoproteins were bound except for aggregated proteins (Fig. 6B). Some intracellular glycoprotein bands of TIG-3S against MAH reacted stronger than that of TIG-102 (e.g. around molecular weights of 245 KDa). These results showed that there was a difference of sialylated glycoprotein expression pattern between TIG-3S and TIG-102 in both intracellular and membrane glycoproteins.

We also analyzed the expression of intracellular and membrane glycoproteins in Gal-binding lectins. Many intracellular glycoproteins of TIG-3S and TIG-102 were bound to WFA, and many membrane glycoproteins were also bound to WFA, except for weakly reacted to membrane glycoproteins of TIG-102 (Fig. 6C). The total amount of intracellular glycoprotein bands of TIG-3S against WFA reacted slightly stronger than that of TIG-102. Also, membrane glycoprotein bands of TIG-3S reacted strongly to WFA compared to that of TIG-102. In binding to ECA, both intracellular glycoproteins were bound, and many membrane glycoproteins were also bound to ECA (Fig. 6D). Although the total amount of intracellular glycoproteins of TIG-3S and TIG-102 against ECA were approximately equal to each membrane glycoprotein taking into account aggregated proteins, both fractionated glycoprotein bands of TIG-102 reacted stronger than that of TIG-3S. These results showed that there was a difference of desialylated glycoprotein expression pattern between TIG-3S and TIG-102 in both intracellular and membrane glycoproteins.

Considering that the band patterns binding to four lectins were different, these results suggest that sialylation to glycoproteins was more pronounced in the intracellular glycoproteins compared to the membrane glycoproteins. Also, these suggest that the expression of α2-6sialylated intracellular glycoproteins was higher in TIG-102 and that of α2-3sialylated intracellular glycoproteins was higher in TIG-102.

## DISCUSSION

Glycans and glycosylated proteins in serum can serve as biomarkers; it was previously reported that WFA-positive Mac-2-binding protein level was elevated in chronic pancreatitis, while the abundance of cancer-specific *N*-glycans on leukemia inhibitory factor receptor (i.e., centrosome-associated protein 350), vacuolar protein sorting-associated protein 13A, and haptoglobin was altered in pancreatic cancer subjects [31,32]. As mentioned above, many intracellular glycoproteins were secreted into serum with various conditions. On the other hand, glycoproteins regulate autophagic machinery, and glycolipids such as lipopolysaccharide are involved in the induction of autophagy in lung tissue [33,34]. These secretory glycans likely regulate molecular function and are serum biomarkers for abnormal glycan metabolism associated with autophagy. We previously analyzed cell surface glycan profiles [10] but did not study intracellular glycan dynamics. Majority of the intracellular glycans are localized in the organelles, such as the endoplasmic reticulum and the Golgi apparatus, and are strictly controlled by glycosyltransferase (Fig. 1A). Many proteins are modified with glycans, move to the cell surface, where they take on specific identities and perform specific functions. The remaining glycoproteins are catabolized and recycled and play an important role in cell survival. This suggests that intracellular glycosylation changes are relatively smaller than that in membrane glycoproteins which are important in distinguishing cell kinds, cell interactions and differentiation. Given that secreted intracellular and membrane glycans can act as regulatory factors, we hypothesized that the relationship between intracellular glycan levels and aging would provide insight into the molecular mechanism of aging, as suggested by the correlation between cell surface glycan expression and cellular senescence or human aging that was reported in our earlier study [10].

In this study, we observed changes in intracellular glycan profiles with cellular senescence (Figs. 1B and 2). When they reached a specific stage (PDL 94), fetus-derived TIG-3S showed signs of aging in PC1 (Fig. 2). TIG-101 and TIG-102 showed a much wider range of values in PC4 in comparison with TIG-3S. We consider that the wider range of values represented a genomic instability associated with the glycan-gene in the elderly or a non-uniformity of intracellular glycoproteins with apoptosis and protein synthesis as shown in Figure 3. Before senescence, TIG-3S and elderly subject-derived TIG-101 and TIG-102 maintained intracellular glycan components and consequently, constant glycan profiles along with their cellular functions (Figs. 2, 3, and Fig. S2). Moreover, quantitative changes in intracellular and membrane glycans were closely correlated with cellular senescence and human aging (Fig. 4). Most of the glycoprotein expression was reflected in abundance of a ratio of intracellular and membrane glycans (Figs. 5 and 6). Glycosylated membrane proteins were exclusively expressed in a part of total membrane proteins that was clear the alteration of sialylation with aging or the existence of sialic acids. In addition. the expression pattern of sialylated intracellular and membrane proteins were different in subject-derived cells (Fig. 6). Cell surface or extracellular glycans can distinguish different cell types such as embryonic stem and embryonal carcinoma cells and contribute to cell function, for instance the regulation of muscular dystrophy [26,35]. The expression of various genes related to glycan synthesis changes markedly depending on cell differentiation state [36,37]. In general, levels of cell surface glycans change gradually, whereas those of intracellular glycans seem to change markedly in a stepwise fashion as a result of cellular alterations that occur during human aging. We propose that not only membrane but also intracellular glycans play an important role in aging.

The abundance of α2-6sialic acid residues of intracellular glycans increased with cellular senescence in total cell extracts, as did that of cell surface Gal residues, which are generated by desialylation (Fig. 4). Cell surface 9-O-acetylated sialic acid (9-O-Ac-sialic acid) bound by a part of influenza virus exists in the Golgi apparatus, too. 9-O-Ac-sialic acid is degraded by 9-O-acetylestrase in the lysosome or cytoplasm, releasing free sialic acid. The de-O-Ac-sialic acid is released in the cytoplasm from the lysosome, followed by recycling. Free sialic acid impacts the abundance of glycan-donor, CMP-Sia. The CMP-Sia synthesized in the nucleus was transfer to proteins by sialyltransferase and formed some glycoconjugates in the Golgi apparatus. As mentioned above, the transport of the sialylated proteins to the cell surface is influenced by O-acetylesterase in the lysosome and the cytoplasm or other glycosyltransferases including sialyltransferase in the Golgi apparatus [38–40]. It is considered that the Golgi enzymes associated with Golgi transport also influence the decline in transport with aging. Therefore, we speculate that there are various enzymatic dysfunctions with aging resulting in the intracellular accumulation of sialylated proteins. It was previously reported that serum levels of sialylated tri- and tetra-antennary complex-type *N*-glycans were elevated in patients with germ cell tumors [41]. Sialylated glycans that accumulate in the cytoplasm with aging may be secreted into the serum under disease conditions. However, we have shown that cell membrane sialidase expression was increased at early stages of cellular senescence [11]. It is thus possible that cell surface desialylation was accelerated by aging-associated glycan accumulation. It is known that cytoplasmic sialic acid level is reduced in hereditary distal myopathy with rimmed vacuoles/hereditary inclusion body myopathy through the suppression of uridine diphosphate-GlcNAc-2-epimerase/*N*-acetylmannosamine kinase activity [42,43]. These findings highlight the importance of sialic acid residue turnover and suggest the possibility of sialic acid replacement therapy.

We, therefore, conclude that qualitative and quantitative alterations in intracellular and membrane glycoprotein are critical for proper cell functioning. Clarifying the correlation between glycan dynamics and aging can provide important insights into the mechanisms of aging as well as a basis for developing therapies for aging-related diseases in the future.

## MATERIALS AND METHODS

### Cell culture

The three dermal skin fibroblast lines from humans were purchased from the Health Science Research Resources Bank (Osaka, Japan). TIG-3S (fetus), TIG-101 (86-year-old subject), and TIG-102 (97-year-old subject) had PDLs of 23, 34, and 29, respectively. Cell proliferative capacity was assessed by calculating total PDL with the formula PDL = log_2_(total number of cells/initial number of cells). PDL counts were rounded up after the decimal point. Cells were maintained in Dulbecco’s Modified Eagle’s Medium (Wako Pure Chemical Industries, Osaka, Japan) containing 10% fetal bovine serum (Cell Culture Technologies, Gravesano, Switzerland), 50 U/ml penicillin, and 50 µg/ml streptomycin (Gibco, Grand Island, NY, USA). Cultures were sub-cultured in 100-mm plastic dishes (BD Falcon, San Jose, CA, USA) at 37°C under a humidified 5% CO_2_ atmosphere. Cell pellets were collected for determination of PDL as previously reported (PDL: TIG-3S, 27–94; TIG-101, 40–51; and TIG-102, 40–52) [10].

### Cell collection and protein extraction

Total protein was extracted from TIG-3S, TIG-101, and TIG-102 cells (approximately 5 × 10^4^ to 1 × 10^6^ cells) collected at various PDLs using CelLytic MEM Protein Extraction kit (Sigma-Aldrich, St. Louis, MO, USA) as previously described [7,10,26]. Briefly, 300 μl cold Lysis Buffer, containing 1% protease inhibitor cocktail was added to the cells, incubated on ice for 10 min, the supernatant was collected and centrifuged at 10,000 *g* at 4°C for 5 min, and was transferred to a new microcentrifuge tube. The supernatant was incubated at 30°C for 5 min and allowed to separate into upper hydrophilic phase and the lower hydrophobic phase. After the supernatant separated, it was centrifuged at 3,000 *g* and 25°C for 5 min, and then the upper and the lower phase solutions were collected separately. Protein concentration was determined using the Micro BCA Protein Assay kit (Thermo Fisher Scientific, Waltham, MA, USA).

### Lectin microarray analysis

Lectin microarray analysis of hydrophilic protein extracts was performed as previously described [10]. Briefly, total proteins including glycoproteins (0.2 μg) were labeled with Cy3 mono-reactive dye (GE Healthcare, Buckinghamshire, UK) in phosphate-buffered saline (PBS) at room temperature for 1 h. The reaction solution was diluted with 20 μl of probing buffer (Tris-buffered saline containing 1% Triton X-100, 1 mM CaCl_2,_ and 1 mM MnCl_2_ [pH 7.4]), and excess dye was removed with a spin-type column loaded with Sephadex G-25 fine matrix (GE Healthcare). The collected Cy3-labeled glycoprotein solution was diluted to 2 µg/ml and the solution (0.25 µg/ml) was applied to a LecChip (v.1.0; Glyco Technica, Yokohama, Japan; Supplementary Table S1). After incubation at 4°C for approximately 17 h, the reaction solution was discarded. The glass slide was washed three times with probing buffer before the LecChip was scanned using the GlycoStation Reader 1200 evanescent-field fluorescence scanner (Glyco Technica). Each sample was measured in triplicate. Data were analyzed using GlycoStation Tools Signal Capture 1.0 and GlycoStation Tools Pro 1.0 (Glyco Technica). To expand the dynamic range, data were subjected to a gain-merging procedure, and merged data were normalized by max-normalization [22].

### Statistical analysis

Lectin microarray data from triplicate measurements were analyzed and displayed using TIGR MultiExperiment Viewer (http://www.tm4.org/mev.html). The values were calculated as log10—i.e., the minimum and maximum values indicate 0.1 and 100, respectively. Data were also evaluated by PCA with pair-wise comparisons (http://lgsun.grc.nia.nih.gov/ANOVA/; false discovery rate < 0.05). The mean value of the lectin microarray data was used for each respective PCA.

### Rate of intracellular or membrane glycan alteration

To evaluate changes in proportions of intracellular or membrane glycans in TIG-3S, TIG-101, and TIG-102, the difference between the signal intensity at each PDL and that at the first PDL was calculated for 45 lectins using the logarithmic formula: ratio = log_2_(signal intensity at each PDL − signal intensity at first PDL). The maximum variance value (∆max) among lectins reflected the maximum difference between the highest value and lowest values at each PDL in each cell line.

### Comparison of glycan abundance

The abundance of each intracellular and membrane glycan recognized by SNA, SSA, ACG, MAH, ECA, PWM, and WFA was determined by calculating the signal intensities of max-normalized data using the formula: percentage abundance = ratio of signal intensity of each lectin in hydrophobic or hydrophilic protein extract/ratio of total signal intensity of hydrophobic and hydrophilic protein extracts.

### Immunocytochemistry with lectin and cellular localization markers

TIG-3S (PDL 38 and 52 for membrane and intracellular, and PDL 48 and 84 for younger and older, respectively), and TIG-102 (PDL 46 and 46 for membrane and intracellular, respectively) were fixed with 4% paraformaldehyde and washed with PBS. To analyze membrane glycan location, the fixed cells were incubated with BlockAid Blocking Solution (Thermo fisher scientific Inc.) at room temperature for 20 min, followed by reaction for 30 min in the dark with a mixture solution, which contained 10 μg/ml biotinylated SNA (Vector Laboratories Inc., Burlingame, CA, USA) and 0.25 μg/ml FITC conjugated CD44 (BD Bioscience, USA) in PBS. The cells were then rinsed three times for 5 min each with PBS, and stained with diluted streptavidin conjugated with Texas Red (StAv-TexRed) (Vector Laboratories Inc.) in PBS (1:100). After rinsing, the cells were stained with DAPI (1:500 in PBS) for 10 min, followed by three 5 min-rinses in PBS, and mounted using Mounting Medium (Dako, Agilent Technologies, Inc., US). To analyze intracellular glycan location, the fixed cells were rinsed with PBS containing 0.1% Triton X-100 (PBSTx) and blocked with BlockAid Blocking solution containing 0.2% Triton X-100 for 20 min at room temperature, followed by reaction for 30 min in the dark with 10 μg/ml biotinylated MRPS27 (Proteintech, USA) in PBS. The cells were then rinsed three times for 5 min each with PBSTx, the cells were stained with a mixture solution, containing a diluted StAv-TexRed and 10 μg/ml FITC conjugated SNA (Vector Laboratories Inc.) in PBSTx.

### Lectin blot analysis of intracellular and membrane glycoproteins

Three μg of the hydrophilic and hydrophobic protein extracts from TIG-3S (PDL 46) and TIG-102 (PDL 49) were applied to each lane of a 5-20% gradient gel for SDS-PAGE. The proteins were transferred to a PVDF membrane and incubated with each of 1 μg/ml biotinylated SNA (EY Laboratories Inc., San Mateo, CA), 2 μg/ml biotinylated WFA (EY Laboratories Inc.), 2 μg/ml biotinylated MAH (Vector Laboratories Inc.) and 2 μg/ml biotinylated ECA (J-OIL MILLS Inc., Tokyo, Japan) at room temperature for 1 h. It was incubated with an HRP-conjugated streptavidin (Jackson Immuno Research Laboratories Inc., USA) at room temperature for 30 min and detected with ECL Prime Western Blotting Detection Reagent (GE Healthcare UK Ltd., England) for 5 min. The proteins were visualized with the Fusion SOLO0.7SEDGE (M&S Instruments Inc., Osaka, Japan).

## Supplementary Material

Supplementary Figures

Supplementary Tables
