# Supplementary material for: Qualitative and quantitative alterations in intracellular and membrane glycoproteins maintain the balance between cellular senescence and human aging
Source: Aging (Albany NY). 2018 Aug 29;10(8):2190–208. doi: 10.18632/aging.101540 (PMC6128432; doi:10.18632/aging.101540)
Supplement: Supplementary Figures [file aging-10-101540-s001.pdf]

SUPPLEMENTARY FIGURES

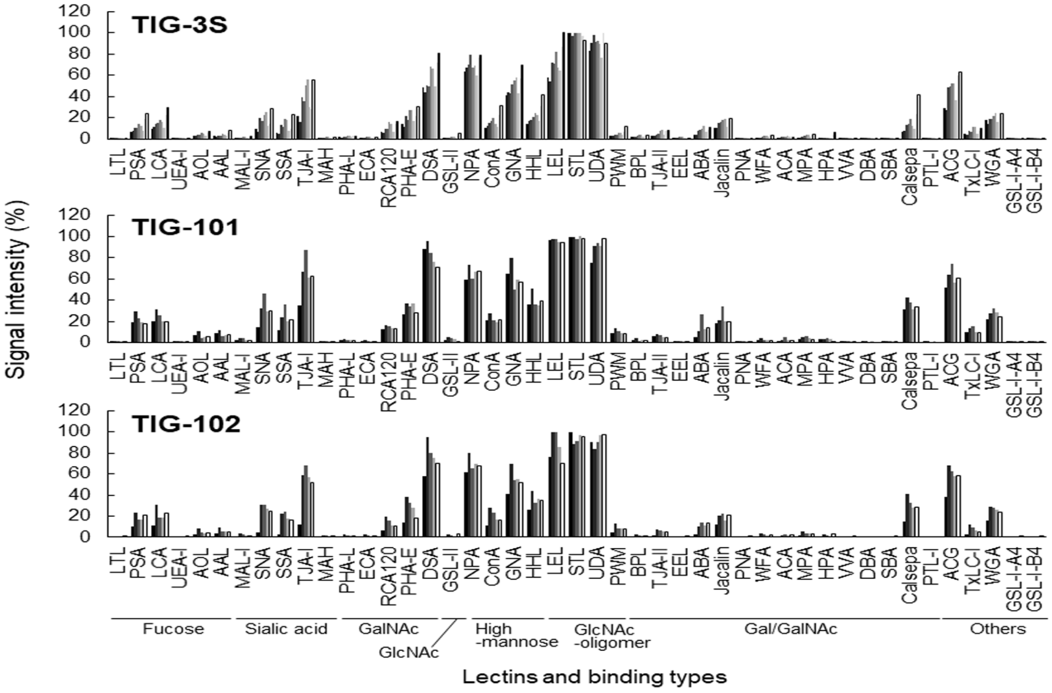

Supplementary Figure S1. Intracellular glycan profiles obtained by lectin microarray analysis in three cell lines (TIG-3S, TIG-101, and TIG-102) at various PDLs. Bar graph representation of signal intensities (%) of 45 lectins in TIG-3S, TIG-101, and TIG-102 at PDL 27–94, 40–51, and 40–52, respectively. Data represent the average of three measurements. Color gradients (dark to light) reflect progressive cellular senescence in PDLs (i.e., young to aged). Characteristics of 45 lectins are listed in Supplementary Table S1 and the value of signal intensities obtained in this analysis are shown in Supplementary Table S2.

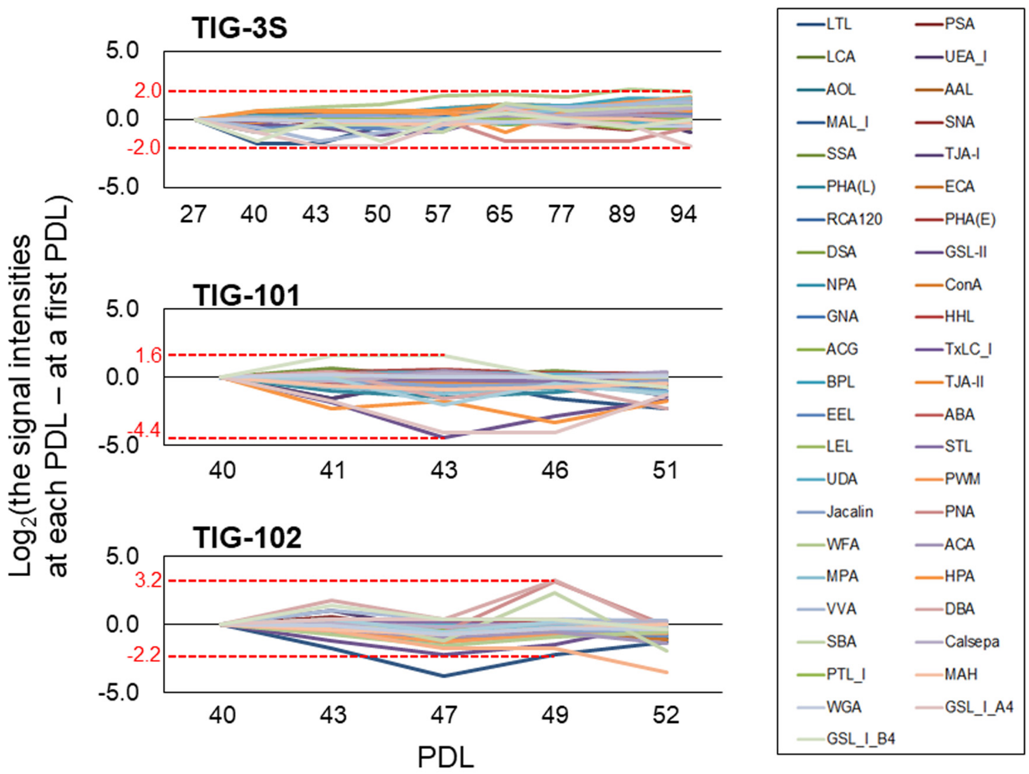

Supplementary Figure S2. Change in ratio of each lectin in cell surface glycans with cellular senescence. Line graph represents differences between lectin signal intensities at various PDLs and those at the first PDL in TIG-3S, TIG-101, and TIG-102. Changes in ratio were calculated from average signal intensity at each PDL. Highest and the lowest values of the largest change in the ratio are shown for each cell line. Each lectin is shown as a different color in a box.

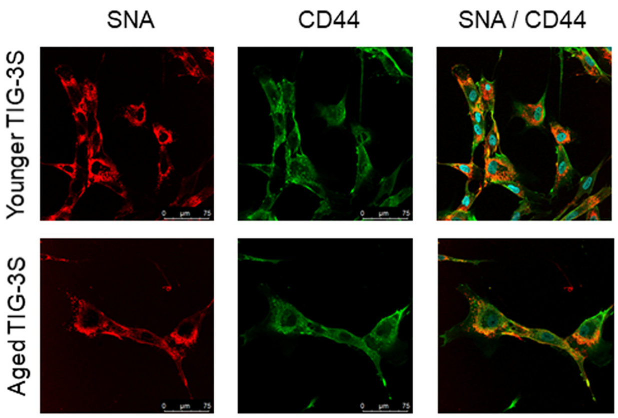

Supplementary Figure S3. Localization of sialylated membrane glycoproteins on TIG-3S. TIG-3S (PDL 48; top and 84; bottom) stained with SNA (red; left panel), FITC-conjugated membrane marker (CD44, green; middle panel), and the overlay image (right panel). Blue staining represents the nucleus.
