# Supplementary material for: Qualitative and quantitative alterations in intracellular and membrane glycoproteins maintain the balance between cellular senescence and human aging
Source: Aging (Albany NY). 2018 Aug 29;10(8):2190–208. doi: 10.18632/aging.101540 (PMC6128432; doi:10.18632/aging.101540)
Supplement: Supplementary Tables [file aging-10-101540-s002.pdf]

SUPPLEMENTARY TABLES

Supplemental Table S1. A List of lectins for microarray.

Abbreviation	Lectin name	Oligosaccharide binding specificity [*1]	Reference No. of LfDB [*2]
LTL	*Lotus tetragonolobus*	Fucα1-3(Galβ1-4)GlcNAc, Fucα1-2Galβ1-4GlcNAc	LfDB0178
PSA	*Pisum sativum*	Fucα1-6GlcNAc, α-Man	LfDB0181
LCA	*Lens culinaris*	Fucα1-6GlcNAc, α-Man	LfDB0171
UEA-I	*Ulex europaeus*	Fucα1-2Galβ1-4GlcNAc	LfDB0185
AOL	*Aspergillus oryzae*	Fucα1-6GlcNAc, Fucα1-2Galβ1-4GlcNAc	LfDB0129
AAL	*Aleuria aurantia*	Fucα1-3(Galβ1-4)GlcNAc, Fucα1-6GlcNAc	LfDB0124
MAL-I	*Maackia amurensis*	Siaα2-3Galβ1-4GlcNAc	-
SNA	*Sambucus nigra*	Siaα2-6Gal/GalcNAc	LfDB0233
SSA	*Sambucus sieboldiana*	Siaα2-6Gal/GalcNAc	LfDB0232
TJA-I	*Trichosanthes japonica*	Siaα2-6Gal/GalcNAc	LfDB0224
PHA-L	*Phaseolus vulgaris*	tri/tetra-antennary complex-type *N*-glycan	LfDB0180
ECA	*Erythrina cristagalli*	Galβ1-4GlcNAc	LfDB0175
RCA120	*Ricinus communis*	Galβ1-4GlcNAc	LfDB0231
PHA-E	*Phaseolus vulgaris*	bi-antennary complex-type *N*-glycan, bisecting GlcNAc	LfDB0179
DSA	*Datura stramonium*	Galβ1-4GlcNAc, (GlcNAc)$_n$	LfDB0162
GSL-II	*Griffonia simplicifolia*	agalactosylated tri/tetra antennary glycans, GlcNAc	LfDB0176
NPA	*Narcissus pseudonarcissus*	Manα1-6Man	LfDB0208
ConA	*Canavalia ensiformis*	Manα1-6(Manα1-3)Man	-
GNA	*Galanthus nivalis*	Manα1-3Man	LfDB0206
HHL	*Hippeastrum hybrid*	Manα1-3Man, Manα1-6Man	LfDB0207
ACG	*Agrocybe cylindracea*	Galβ1-3Gal, Siaα2-3Galβ1-4Glc	LfDB0125
TxLCI	*Tulipa gesneriana*	Manα1-3(Manα1-6)Man, bi-antennary complex-type *N*-glycan	-
BPL	*Bauhinia purpurea alba*	Galβ1-3GalNAc	LfDB0173
TJA-II	*Trichosanthes japonica*	Fucα1-2Galβ	LfDB0225
EEL	*Euonymus europaeus*	Galα1-3Galβ1-4GlcNAc, Fucα1-2Galβ1-3GlcNAc	LfDB0222
ABA	*Agarics bisporus*	Galβ1-3GalNAc	LfDB0126
LEL	*Solanum lycopersicum*	(GlcNAcβ1-4)$_n$, (Galβ1-4GlcNAc)$_n$	LfDB0164
STL	*Solanum tuberosum*	(GlcNAcβ1-4)$_n$	LfDB0167
UDA	*Urtica dioica*	(GlcNAcβ1-4)$_n$, High-Man	LfDB0168
PWM	*Phytolacca americana*	(GlcNAcβ1-4)$_n$	LfDB0165
Jacalin	*Artocarpus integrifolia*	Galβ1-3GalNAc, GalNAcα, GlcNAcβ1-3GalcNAc, Manα1-6(Manα1-3)Man	LfDB0146
PNA	*Arachis hypogaea*	Galβ1-3GalNAc	LfDB0172
WFA	*Wisteria floribunda*	GalNAcβ1-4GlcNAc, Galβ1-3GalNAc	LfDB0188
ACA	*Amaranthus caudatus*	Galβ1-3GalNAc, Siaα2-3Galβ1-3GalNAc	-
MPA	*Maclura pomifera*	GalNAcα, Galβ1-3GalNAc	-
HPA	*Helix pomatia*	GalNAcα	LfDB0105
VVA	*Vicia villosa*	GalNAcβ1-4Gal, GalNAcβ1-3Gal, GalNAcα	LfDB0187
DBA	*Dolichos biflorus*	GalNAcα1-3(Fuca1-2)Galβ, GalNAcα1-3GalNAc	LfDB0174
SBA	*Glycine max*	Tarminal GalNAc	LfDB0166
Calsepa	*Calystegia sepium*	Galactosylated bi-antennary complex-type *N*-glycan with bisecting GlcNAc, High-Man	LfDB0156
PTL-I	*Psophocarpus tetragonolobus*	GalNAcα, Galα1-3(Fucα1-2)Galβ	LfDB0182
MAH	*Maackia amurensis*	Siaα2-3Galβ1-3GalNAc	-
WGA	*Triticum vulgaris*	(GlcNAcβ1-4)$_n$, Hybrid type *N*-glycan with bisecting GlcNAc	LfDB0163
GSL-I-A_4	*Griffonia simplicifolia*	GalNAcα	LfDB0229
GSL-I-B_4	*Griffonia simplicifolia*	Galα	LfDB0230

Each lectin was showed as abbreviation, lectin name and main specificity on LecChip (ver. 1.0).
[*1] This was modified of Glyco Technica Ltd. web list. [*2] LfDB is shown from web site (http://acgg.asia/lfdb2/index).

Supplemental Table S2. Lectin microarray data of TIG-3S, TIG-101 and TIG-102.

	TIG-3S (%)									TIG-101 (%)					TIG-102 (%)			
Lectin / PDL	27	40	43	50	57	65	77	89	94	40	41	43	46	51	40	43	47	49
LTL	0.5	0.5	0.6	0.7	0.8	0.9	0.6	0.7	0.5	0.4	0.7	0.5	0.6	0.3	0.0	0.5	0.5	0.2
PSA	7.0	7.7	10.8	10.2	14.2	12.6	7.6	18.5	24.3	19.0	29.7	22.6	18.9	17.4	9.9	23.7	16.4	17.1
LCA	9.8	10.9	14.2	15.0	17.5	16.0	9.9	22.4	29.6	20.4	31.5	25.2	20.1	19.4	11.5	30.8	18.5	18.6
UEA-I	0.5	0.4	0.6	0.7	0.8	0.7	0.4	0.6	0.3	0.4	0.4	0.2	0.4	0.1	0.0	0.5	0.3	0.1
AOL	2.7	2.8	3.5	4.1	5.7	4.8	2.9	5.7	6.9	7.2	10.7	4.3	5.0	5.2	2.5	8.2	5.0	4.2
AAL	2.5	2.4	2.7	3.3	4.9	4.3	2.7	5.9	7.6	8.5	12.0	6.3	7.0	7.4	3.4	9.6	5.8	5.2
MAL-I	0.7	0.6	1.1	1.1	1.8	2.1	0.9	1.9	2.3	2.0	4.0	3.8	2.3	1.9	0.5	3.5	2.6	1.8
SNA	9.8	7.0	19.2	17.0	22.6	25.3	12.5	13.7	28.5	14.8	32.4	46.2	29.1	29.8	4.5	30.4	31.1	26.6
SSA	6.0	4.6	12.8	11.2	18.3	17.8	8.0	9.8	22.5	11.2	23.7	36.2	20.3	21.6	2.8	22.0	24.7	17.6
TJA-I	21.8	16.1	38.9	35.8	50.4	55.6	30.3	28.5	55.4	35.0	66.7	87.2	60.6	62.2	12.1	59.0	68.1	56.5
PHA-L	1.5	1.4	2.0	1.8	2.8	3.1	1.8	3.1	2.4	2.0	3.4	2.2	2.3	1.5	0.7	3.1	2.3	1.6
ECA	1.0	1.0	1.5	1.6	2.1	2.0	1.2	2.0	1.6	1.3	2.1	1.3	1.5	1.0	0.5	2.5	1.7	1.0
RCA120	6.3	5.9	9.8	9.1	15.7	14.0	7.0	14.3	16.0	12.8	16.6	15.7	13.3	12.6	6.3	19.5	15.5	11.5
PHA-E	14.5	11.5	21.8	18.2	27.3	27.4	17.3	21.8	30.2	26.6	36.7	34.3	36.6	27.6	14.0	38.1	32.6	28.3
DSA	48.8	43.7	50.8	49.5	68.4	66.4	49.1	71.4	80.8	88.2	95.4	84.1	76.1	71.0	57.8	94.8	79.8	75.1
GSL-II	0.9	0.6	1.4	1.3	1.7	2.3	1.0	3.3	4.8	2.7	4.7	4.2	3.4	0.5	0.8	2.4	1.8	1.2
NPA	63.6	66.8	70.2	79.2	67.0	69.5	59.6	76.2	78.8	58.9	73.5	59.8	67.0	67.4	61.9	80.4	65.4	69.5
ConA	10.0	11.8	15.1	16.7	19.7	13.9	11.3	22.4	31.0	20.5	27.6	20.7	18.9	21.6	11.1	28.1	23.4	17.2
GNA	41.0	43.7	42.7	51.2	55.2	57.5	43.3	56.6	69.3	64.4	79.9	50.3	59.1	56.4	40.7	69.9	54.0	55.0
HHL	14.2	16.6	17.9	20.3	24.5	22.5	17.1	31.2	41.7	36.2	51.1	35.7	34.8	39.0	25.8	44.2	32.5	36.1
ACG	29.4	26.7	48.7	49.1	52.2	51.8	36.7	60.0	63.4	51.7	63.4	74.0	56.1	60.9	38.3	68.1	62.6	57.8
TxLC-I	4.8	3.8	7.4	6.5	11.2	11.6	4.3	7.8	10.3	9.8	13.8	15.5	9.5	8.9	2.8	12.3	9.5	6.8
BPL	1.6	1.5	1.9	2.4	3.5	3.5	1.9	3.5	3.1	2.6	3.9	1.8	2.1	1.6	0.8	3.1	2.3	1.8
TJA-II	3.3	3.0	4.1	4.9	8.0	8.4	3.8	7.2	7.6	5.6	8.1	6.6	5.0	4.6	2.1	7.6	6.2	5.5
EEL	1.1	1.1	1.4	1.6	1.9	2.2	1.2	1.5	0.6	0.9	1.7	0.9	1.1	0.4	0.2	1.2	1.0	0.6
ABA	3.8	3.5	7.1	8.8	9.3	11.8	6.5	8.8	10.7	5.6	10.8	26.4	12.2	13.5	2.8	10.0	14.4	10.8
LEL	57.8	54.2	72.3	71.0	82.3	66.8	64.0	86.7	100.0	96.6	97.2	97.5	94.8	93.9	76.8	99.5	100.0	85.8
STL	100.0	100.0	97.0	100.0	100.0	100.0	100.0	96.7	92.4	99.3	99.0	97.7	100.0	97.6	100.0	88.2	91.2	96.9
UDA	83.2	90.0	98.1	91.0	92.7	89.8	76.5	99.6	89.7	75.0	91.1	93.3	91.0	98.3	90.4	83.7	90.3	97.3
PWM	3.1	3.1	3.8	4.0	6.0	5.7	3.8	6.9	11.4	8.6	13.9	10.3	8.7	8.8	4.7	12.7	8.6	8.5
Jacalin	10.3	10.3	15.4	15.9	18.0	18.4	11.6	19.2	19.5	18.0	21.4	33.7	20.1	19.5	11.8	20.7	22.5	16.2
PNA	0.4	0.3	0.3	0.4	0.5	0.7	0.4	0.4	0.1	0.3	0.5	0.1	0.4	0.0	0.0	0.3	0.2	0.1
WFA	1.0	0.9	1.3	1.7	3.3	3.2	1.6	3.8	3.6	2.5	3.8	2.1	1.9	1.9	1.0	3.9	2.9	2.3
ACA	1.3	1.3	2.0	2.0	2.3	2.9	1.6	2.3	1.9	1.4	2.7	5.5	2.6	2.3	0.6	2.2	3.0	1.8
MPA	1.4	1.4	1.9	2.5	3.5	3.6	2.0	3.7	4.2	3.0	5.4	5.7	4.0	2.8	1.7	5.3	3.9	3.3
HPA	0.6	0.1	1.2	0.9	1.2	1.2	0.3	3.8	5.9	2.9	3.5	4.6	3.5	0.3	0.7	2.7	1.8	0.8
VVA	0.5	0.3	0.3	0.4	0.6	0.6	0.5	0.7	0.5	0.4	0.8	0.2	0.5	0.2	0.0	0.6	0.3	0.2
DBA	0.3	0.2	0.2	0.2	0.2	0.3	0.3	0.3	0.1	0.0	0.1	0.0	0.2	0.0	0.0	0.1	0.1	0.0
SBA	0.4	0.4	0.4	0.6	0.7	0.8	0.4	0.9	0.7	0.4	0.7	0.3	0.5	0.3	0.1	0.5	0.3	0.2
Calsepa	7.0	7.6	13.5	14.4	19.0	12.5	9.8	23.0	41.7	31.6	42.0	37.7	31.2	33.4	15.1	41.0	32.5	28.1
PTL-I	0.4	0.3	0.3	0.4	0.6	0.7	0.3	0.3	0.0	0.2	0.3	0.0	0.3	0.0	0.0	0.2	0.1	0.0
MAH	1.3	1.1	1.4	1.5	2.0	2.1	1.1	1.8	1.1	1.0	1.7	1.4	1.4	0.7	0.2	1.6	1.6	0.8
WGA	17.7	14.4	18.9	18.3	21.6	24.5	16.2	24.6	24.1	21.6	27.4	31.8	28.3	24.2	15.5	29.3	27.6	25.8
GSL-I-A4	0.3	0.3	0.5	0.4	0.7	0.7	0.4	0.5	0.5	0.5	1.2	0.9	0.9	0.4	0.0	1.0	0.7	0.3
GSL-I-B4	0.8	0.7	0.7	0.8	1.0	1.1	0.7	0.8	0.2	0.3	0.6	0.2	0.6	0.1	0.0	0.5	0.4	0.1

Each cell at the indicated population doubling level (PDL) was applied for lectin microarray analysis. The data was averaged at each PDL after normalization (n =3).

Supplemental Table S3. A ratio of intracellular and membrane glycans for total cell extractions.

	TIG-3S									TIG-101					TIG-102				
**Lectin / PDL**	**27**	**40**	**43**	**50**	**57**	**65**	**77**	**89**	**94**	**40**	**41**	**43**	**46**	**51**	**40**	**43**	**47**	**49**	**52**
**Intracellular glycans / total extractions (%)**																			
SNA	42	37	55	58	58	56	54	55	55	62	76	74	73	69	41	72	76	71	68
SSA	36	31	50	50	56	52	47	45	49	56	72	72	68	64	28	64	72	61	56
ACG	34	33	39	41	40	45	42	54	54	60	52	52	52	56	58	55	48	55	55
MAH	39	37	38	43	47	44	35	39	30	11	20	23	17	13	3	16	20	11	15
ECA	45	39	41	45	39	34	32	32	24	13	22	18	20	13	6	21	23	11	11
PWM	60	60	62	59	59	56	52	50	53	38	47	39	41	39	34	49	46	43	40
WFA	31	21	20	23	22	21	18	21	20	17	28	17	18	12	10	26	23	16	13
**Membrane glycans / total extractions (%)**																			
SNA	58	63	45	42	42	44	46	45	45	38	24	26	27	31	59	28	24	29	32
SSA	64	69	50	50	44	48	53	55	51	44	28	28	32	36	72	36	28	39	44
ACG	66	67	61	59	60	55	58	46	46	40	48	48	48	44	42	45	52	45	45
MAH	61	63	62	57	53	56	65	61	70	89	80	77	83	87	97	84	80	89	85
ECA	55	61	59	55	61	66	68	68	76	87	78	82	80	87	94	79	77	89	89
PWM	40	40	38	41	41	44	48	50	47	62	53	61	59	61	66	51	54	57	60
WFA	69	79	80	77	78	79	82	79	80	83	72	83	82	88	90	74	77	84	87

Ratios of each seven lectin signal were showed in TIG-3S, TIG-101 and TIG-102 at the population doubling level (PDL). The data was calculated by averaged signal intensities (n = 3).
